# Examining the Determinants of Healthcare Workers’ Performance: A Configurational Analysis during COVID-19 Times

**DOI:** 10.3390/ijerph18115671

**Published:** 2021-05-25

**Authors:** Benito Yáñez-Araque, Sagrario Gómez-Cantarino, Santiago Gutiérrez-Broncano, Víctor-Raúl López-Ruiz

**Affiliations:** 1 Department of Physical Activity and Sports Sciences, Applied Intelligent Systems Research Group, University of Castilla-La Mancha, Av. Carlos III, s/n, 45071 Toledo, Spain; 2 Department of Nursing, Physiotherapy and Occupational Therapy, Faculty of Physiotherapy and Nursing, University of Castilla-La Mancha, 45004 Toledo, Spain; Sagrario.Gomez@uclm.es; 3 Department of Business Administration, Faculty of Social Sciences, University of Castilla La Mancha, 45600 Talavera de la Reina, Spain; Santiago.Gutierrez@uclm.es; 4 Department of Spanish and International Economics, Econometrics and History and Economic Institutions, Faculty of Economics and Business, University of Castilla-La Mancha, 02071 Albacete, Spain; Victor.Lopez@uclm.es

**Keywords:** COVID-19, healthcare workers, job performance, leadership, organizational commitment, job satisfaction, work environment, fsQCA

## Abstract

The evaluation of the work performance of health professionals has focused the interest of scientific research in recent decades as a basis for improving the quality of health services. The global COVID-19 pandemic has pushed countries’ health systems to the limit and had previously unknown consequences on the job performance of health professionals. In this context, what are the determinants of performance? There are numerous studies that link job performance with other variables that directly affect it, such as leadership, job satisfaction, organizational commitment, and work environment. However, there are no studies that jointly relate all these variables, and even less in the field of health. The main objective of this work is to analyse how these variables are configured together to generate a good level of performance of health professionals during the times of COVID-19. To do this, a fuzzy set qualitative comparative analysis (fsQCA) is carried out, an appropriate method that will allow finding the joint causal effects of key variables in human resources to ensure a good level of job performance in health organizations. The study reveals that leadership and commitment are the two key drivers of performance. The data confirm that the “recipe” to achieve a good level of performance consists of the combination of leadership, commitment, and a good work environment. Additionally, in the case of less satisfied workers, linking leadership and commitment is a sufficient condition.

## 1. Introduction

The global employment situation of health workers caused by the COVID-19 pandemic requires a lot of attention and concentration, high responsibility, work overload, and long or disorderly hours and shifts; which puts job performance and its health, social and economic repercussions at risk. The pandemic has affected health-care workers’ psychological and mental health [1]. This situation implies special attention to the human side of management. Failure to do so could lead to the effects of bad leadership. Exercising leadership incorrectly can generate inefficiency in the organization, a bad work environment and problems for employees in relation to their motivation, satisfaction and commitment. Additionally, thus cause low productivity, absenteeism and occupational risks such as stress or burnout syndrome, in short, decisively affect the performance of workers.

In the 1980s, the World Health Organization began to study the need to evaluate the performance of health workers, creating the first definitions of job performance in the health field [2]. At present, there is a tendency to accept that the factors that condition performance are related to factors related to the quality of the service. In this sense, Salas et al. [3] define work performance as the behaviour or conduct of workers both in the professional and technical order, as well as in the interpersonal relationships that are created in the care of the health/disease process of the population, which in turn has an important influence on the environmental component. Therefore, there is a direct correlation between the factors that characterize professional performance and those that determine the overall quality of health services.

The analysis of performance quality involves taking into account the working and personal conditions that workers have, as they can influence job performance both positively and negatively. Health-worker practices are complex behaviours that have many potential influences. Inadequate health-worker performance is a very widespread problem [4]. Below, we examine a selection of work variables that are associated in the literature with job performance to the extent that they positively enhance the compatibility of workers in their organisation. These variables are: leadership, organisational commitment, job satisfaction and work climate.

### 1.1. Leadership and Job Performance

Leadership has been widely discussed in the literature. It is an interaction between two or more members of a group that often involves structuring or restructuring the situation and the perceptions and expectations of the members [5]. Leaders are change agents, people whose actions affect other people more than their actions affect them. Leadership occurs when one group member changes the motivation or competencies of other group members. For Suedfeld [6], the social atmosphere of the group in a work situation influences the relationship between leadership style and effectiveness, without forgetting the particular situations within the environment. A person may be an effective leader in one group, but may not be as effective in another. This is due to multiple factors such as the characteristics of the group or the objectives to be achieved. Moreover, within the same group, situations may also vary. Therefore, to be a successful leader, one must have the great ability to become a situational leader, that is, to be able to adapt to changing situations. We call it this because of the ability the leader must possess to use leadership styles as situations change [7]. This was also stated by Schein [8] and Suedfeld [6] highlighting that the leadership style must adapt to the situation and effectiveness, depending on the leader’s ability to do so. Schein [8] reported that, historically, leadership was believed to be a psychological trait and a set of characteristics within the individual. Brown [9] identified leadership qualities as intelligence and good judgment, insight and imagination, ability to accept responsibility, sense of humour, well-balanced personality and sense of justice. According to the latter author, the leader’s abilities include the ability to coordinate, the ability to express the common goal, the ability to delegate, the ability to reflect the progress of the group and impartiality. The leader may be in a position to inspire his subordinates and respond to the needs, concerns, wishes, and desires of the group. On the contrary, the leader could create a demotivating environment that results in an unproductive organization.

In addition to leader qualities, there are different leadership styles that are significantly related to workers’ job performance [10]. Schein [8] showed that democratic styles of supervision led to higher performance and job satisfaction and that social relationships within groups affected productivity. Additionally, social factors, such as relationships with peers and supervisors, were important in job satisfaction; it was widely believed that job satisfaction correlated with, or was the cause of, high productivity. However, later research showed that, with higher job satisfaction, the direct effects of increased productivity were modest (10–25%), although there was lower absenteeism, job turnover and better communication and cooperation. Mendo and Ortiz [11] stated that satisfaction refers to the degree to which people approve of the various aspects of leadership and the results achieved by the team over a given period of time. Hassan et al. [12] argued that ethical leadership reduced absenteeism and had a positive influence on organizational commitment and willingness to report ethical problems. Evkall and Ryhammar [13] pointed out that the main problem for effective performance is the difficulty of providing good leadership. This is corroborated by Robbins and Judge [14], who mentioned that leadership is a process of influence, in which the manager, through his actions, favours the movement of staff towards a common goal or objective, thus being fundamental to lead workers to exercise better work performance. This idea expresses the influence of leadership on a worker’s job performance, where it follows that the area manager has a positive or negative influence on the performance of workers, both individually and collectively.

Therefore, on the relationship between leadership and job performance, we generate the following proposition:

**Proposition** **1.** *Leadership affects job performance.*

### 1.2. Organisational Commitment and Job Performance

Organisational commitment has been related both theoretically and empirically to multiple concepts: attendance, job performance on the job or citizenship behaviour at work [15]. Organisational commitment was originally defined as the strength of an individual to identify with and become involved in a particular organisation. Job performance is characterised by: (a) a strong belief in the acceptance of an organisation’s goals and values; (b) a willingness to exert considerable effort on behalf of the organisation; and (c) a definite desire to maintain organisational membership [7].

Davis and Newstrom [16] pointed out that organisations must have people and individuals trying to achieve a goal. The person in charge of the workers should be aware of the different needs and desires of the people in their charge and integrate them efficiently, seeking to match people with work that demands and inspires their motives and capabilities. Leite et al. [17] stated that the most frequent and most significant commitments in the prediction of this variable are those related to work experiences, such as work characteristics (scope, challenge and variety of tasks), perceptions of fairness, organisational support and established relationships (interdependence of tasks, communication with the leader, participative leadership, among others). Variables related to conflict and role ambiguity also show strong but negative relationships with work engagement. Figueroa et al. [18] distinguished three types of work engagement: affective engagement, continuous engagement and normative engagement. The first, affective commitment, refers to the degree of the employee’s emotional attachment to the organisation and involvement in the organisation’s activities and projects. Affective commitment is related to lower levels of absenteeism and higher levels of performance and citizenship behaviours. Continuous commitment refers to employees’ awareness of the costs associated with eventually leaving the organisation and is characteristic of those whose main motive is that their calculation of the economic conditions offered by the organisation indicates that these are better than the other available options. Finally, normative commitment is based on feelings of employee obligation to stay with the organisation out of gratitude, loyalty, or moral value.

Morrow [19] highlighted the emphasis on the management of affective commitment, since this base represents the most significant relationships with desirable behaviours. Vandenabeele [7] argued that both normative and affective commitment will be linked to performance, while permanence commitment will not be related, or even negatively affect performance. Intuitive and theoretical engagement can easily be related to performance [20], although this relationship was empirically less supported [21,22]. Authors such as Arciniega [23] argued that employees with a high level of commitment will also present better performance, productivity, and lower absenteeism rates.

With this background, we state the following proposition:

**Proposition** **2.** *Job performance improves with organisational commitment.*

### 1.3. Job Satisfaction and Job Performance

Another variable widely discussed in the human resources literature is job satisfaction. It is related to many variables of great importance in productivity and performance, job stress, burnout, absenteeism and turnover, among others. Gruneberg [24] defined job satisfaction as a pleasure or positive emotional state resulting from the evaluation of work or work experiences. Pérez and Fidalgo [25] argued that there are variables that influence job satisfaction by conditioning the affective response to different aspects of work. These variables are the circumstances and characteristics of the job itself and the individual characteristics of each worker. These two variables are used to determine the thresholds of job satisfaction or dissatisfaction. These thresholds, in turn, are conditioned by another series of factors such as the worker’s own personal and professional history, age or sex, training, aptitudes, self-esteem or cultural and socio-economic environment.

Petty et al. [26] concluded that job satisfaction and job performance are positively correlated. These findings were echoed by Judge et al. [27], who found that previous studies were sometimes too restrictive in their conceptualisations of job satisfaction and performance. With respect to the direction of the satisfaction-performance relationship, it was suggested that positive emotions, such as feelings of satisfaction, would produce higher performance. Ultimately, improving job performance increases organisational productivity. Encouraging participative management and providing opportunities for employees to participate in decision making improves employees’ confidence, self-recognition, realisation of their values leading to increased job satisfaction. Basically, job satisfaction increases job performance and job performance depends on employee participation, commitment, and retention. We can say that as job satisfaction increases, absenteeism and turnover decrease [7]. Although it may seem a reason to claim that satisfied employees would perform better, this claim has remained relatively unsupported [28]. In this regard, Spencer and Steers [29] stated that the fact that workers are satisfied does not mean that they will produce more, only that they are satisfied. Careful analysis of the research indicates that, assuming a positive relationship between satisfaction and productivity, the correlation is generally low. However, the inclusion of moderating variables has increased the correlation. For example, the relationship is stronger when employee behaviour is not subject to limits or controls from external factors.

The proposition to test job satisfaction’ impact on job performance derives from literature being inconclusive:

**Proposition** **3.** *Job satisfaction does not ensure better job performance.*

### 1.4. Work Climate and Job Performance

Work climate is one of the most subtle and complex aspects of human resource management, the study and analysis of which has become increasingly important. Litwin and Stringer [30] define it as a property of the organisational environment as described by its members. In this sense, organisational climate is the product of subjective effects perceived by workers with respect to the formal system in which they operate, the informal style of managers and organisational factors (work characteristics, employment conditions, etc.). These factors affect people’s attitudes, beliefs, values, and motivation.

Some characteristics of work climate can be found in the literature. Batlis [31] considers it as an intervening variable influenced by organisational characteristics such as leadership style and specific work activities. This variable, in turn, influences individual performance and attitudes towards work. Perceptions of organisational climate are not evaluations of environmental events and conditions, but descriptions of them. Brunet [32] considers that different group properties contribute to organisational climate (leadership, norms, roles, group cohesion, group processes and group structure) along with organisational processes (performance appraisal, decision making, etc.). Scheneider et al. [33] define it as the atmosphere perceived by employees, which has been created by the organisation’s typical practices, procedures and rewards. The influence of work climate on performance seems to be logical since employees’ perceptions of their work environment condition the way in which the task is structured, the reward system, type of communication, etc.

Some meta-analyses [34] point to dimensions of work climate that are related to job performance, such as supervisory styles, support, risk, decision-making, rewards and peer relations. Silva [35] pointed out that climate is simply a useful tool for understanding and improving performance. This has led authors to speak of the potential impact of climate on performance as an indirect determinant of performance, without establishing a causal relationship between the two variables [36]. Toro and Cabrera [37] stated that climate regulates commitment, motivation, satisfaction, people’s performance at work and the company’s productivity, not as a direct causal agent, but as a facilitating or restrictive environmental reality. Olsen et al. [38] found that the majority of work climate characteristics are confirmed to influence workplace bullying, and additionally have direct influence on nurse outcomes (job performance). Liu and Chiu [39] argued that a perceived benevolent climate positively moderates the relationship between job stress and job performance. Rodriguez et al. [40] pointed out that the literature is often cautious when trying to express the predictive value that climate has on performance. They note that there is a significant relationship between climate, satisfaction, and performance. Considering the dimensions of performance, on the one hand, climate significantly predicts official behaviour and personal conditions, while satisfaction only predicts performance and productivity.

The work environment relates to job performance, an idea which the following proposition tests:

**Proposition** **4.** *Work climate influences job performance.*

### 1.5. Determining Configurations of Job Performance

As we have been able to analyse in the previous sections, numerous studies on the aforementioned work variables relate, for example, leadership with the work climate, or with satisfaction, or employee commitment to the organisation and performance. Although authors such as Rodriguez et al. [40] have already pointed out that performance is better predicted by the variables as a whole, there is a gap in the literature regarding the treatment of all these variables as a whole, and even more so in the healthcare sector. For this reason, the purpose of this study is to analyse how these work variables, leadership, job satisfaction, organisational commitment and perception of work climate are configured to generate a good level of performance in healthcare workers. The research objective is to identify the causal combinations that lead to a good performance in healthcare workers.

**Figure 1:**
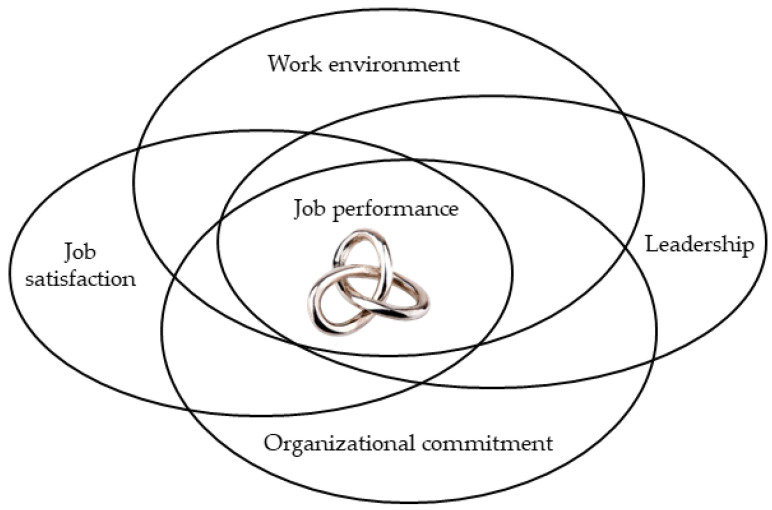
Conceptual model of job performance.

Figure 1 illustrates the interactions between all variables by intersecting orbits, to draw attention to the dynamic mutual interdependence of all elements. The entangled Gordian knot at the centre helps illustrate the notion that the five core elements are closely fused [41].

An fsQCA analysis of these job variables is conducted on a sample of healthcare workers at the National Hospital for Paraplegics. The most significant findings are that leadership and commitment are the key variables to ensure a good level of job performance in the case of less satisfied workers, and, in general, that there is a good work climate, in addition to leadership and commitment.

The following sections present the methodology used to carry out the analysis in accordance with the research objectives. This is followed by the results and discussion, where the findings, limitations and future lines of research will be presented. Finally, the main conclusions are presented.

## 2. Materials and Methods

### 2.1. Participants

The sample selected for the study came from healthcare workers at the National Hospital for Paraplegics, located in the Spanish city of Toledo. It is the public hospital of reference in Spain for the treatment of spinal cord injury. The centre was inaugurated in 1974. After being managed by the former INSALUD (National Health Institute, which was the public entity in charge of health provision and management in Spain until the current National Health System was set up), on 1 January 2002, the Government of Castilla-La Mancha took over the ownership of the National Hospital for Paraplegics. Its scope of action is that of a national reference centre specialising in spinal cord injury, recognised by the Ministry of Health of the Spanish Government. It has an installed capacity of 210 beds, 1 operating theatre, 10 consultation rooms in the Hospital, radiodiagnostic equipment, 3 urodynamics rooms, 2 electrophysiology rooms, and a staff of more than 700 workers. During the first wave of the crisis caused by the coronavirus, the National Hospital for Paraplegics set up three of its floors for admissions with COVID-19 derived from other centres. After the decrease in cases, these floors were disinfected and have been maintained as a reserve area in case of a resurgence. In the face of the second wave of coronavirus, since mid-September 2020, beds have been made available again in this hospital due to the increased pressure of care in other hospitals.

Data collection took place between October and November 2020, at the height of the second wave of coronavirus in Spain. The questionnaires were delivered face-to-face to workers, with the collaboration of supervisors and hospital management. Respondents were assured that their answers were anonymous and that there were no right or wrong answers, and were encouraged to answer the questions as honestly as possible. The questionnaires were completed without the presence of outsiders, to avoid conditioning the worker. Subsequently, a place was set up where the completed questionnaires could be deposited within two weeks. Two hundred and fifty questionnaires were delivered, and for fsQCA, the sample comprises 40 valid cases. These cases contain no missing values for the variables under study. Table 1 lists the characteristics of the sample.

### 2.2. Measurement of Variables

All selected variables were measured based on scales validated in previous studies. For the purpose of the study, all scales were adapted to 7-point Likert or response levels for all indicators, following the recommendations of authors such as Taherdoost [42]. Items are averaged to obtain scores ranging from 1 = strongly disagree to 7 = strongly agree.

Job performance, which is the dependent variable or outcome, was measured based on Vandenabeele’s [7] scale, consisting of four items of perceived performance. This variable is a self-reported performance measure. Such measures are regularly used within public administration [43], as is the case of this study. The following scales were used to measure the independent variables: (a) six-item scale by Samson and Terziovski [44] for leadership; (b) job satisfaction used the six-item scale by Depré and Hondeghem [45]; (c) job commitment used the scale by Benkhoff [46] through the “skeleton” version of the Organizational Commitment Questionnaire, which consists of six items; d) and finally, for work climate, the Organizational Climate Measure (OCM) by Patterson et al. [47], which consists of 17 scales, divided into four quadrants: human relations, internal process, open systems, and rational goal.

Cronbach’s alpha coefficient [48] remains the most widely used statistic of the internal consistency or reliability today. However, for an appropriate and accurate parametric analysis with this coefficient, sample sizes of more than 250 subjects are required [49,50,51]. The same is true for examining the comparisons between them [50,51], which depend on the size of the difference to achieve an acceptable degree of statistical power [52,53], and the sample size to ensure that the asymptotic theory of the distribution of the statistic is correct [54]. Appendix A reports the α coefficient values obtained in the sample together with the authors of the validated scales.

### 2.3. Data Analysis

Following Yáñez-Araque et al. [55], the fsQCA method overcomes the limitations largely attributable to the problems inherent to multiple regression analysis (MRA) and structural equation modelling (SEM) [56,57]: symmetrical causal relationships, net effects, etc. FsQCA is a useful qualitative method for analysing social phenomena with small data sets, allowing for good uncertainty management [58,59]. FsQCA is used for configurational and causal analysis, where different constellations of variables cause different outcomes. For these reasons, the use of fsQCA in this research is appropriate. Our approach has been developed in the same way as El Sawy et al. [41] to show a configurational analysis based on fsQCA. These authors in turn follow Ragin’s recommendations [59] (Chapter 11). We are trying to demonstrate the influence of each variable on the outcome variable.

In recent years, the adoption of qualitative comparative analysis (QCA), in its three versions of csQCA, fsQCA, and mvQCA, has been growing, substituting traditional correlation methods to establish causal conditions related to a particular result. A bibliometric analysis shows the exponential growth since 2007, an increasing trend in the use of fsQCA at the expense of csQCA due to the advantages offered by the former regarding the inclusion of conditions with a degree of membership in the set. This fact allows the prediction that, as new applications are discovered for QCA in other fields, the amount of research and its impact will increase [60]. FsQCA has not been used previously in medical diagnosis [61] and enables us to address an important gap in the current scholarly literature on public management reforms, associated with perceptions of improvements in healthcare efficiency [62].

Up until now Ragin’s approach has been used mainly for the analysis of small datasets. Qualitative Comparative Analysis [58] is a useful method for studying social phenomena when the number of cases is small, that is, a number of cases (*n*) “medium”, it would be very high to treat the case study method, but at the same time, very low for statistical analysis. Even if we can apply fuzzy sets for small *n* studies, its relevance is not evident. On the same way, applying QCA (cs/mv) to large *n* comparison, increases the probability of “contradictory” cases and so decreases the relevancy of the method as to determine “conditional configurations”. Fuzzy sets’ optimal application correspond to a “moderate” (>6 cases) to “relatively high” *n* of cases (>25 cases). Therefore, the sample size of the study is adequate.

The data analysis tool used was the FsQCA 2.5 software (University of California, Irvine, CA, USA) [63].

## 3. Results

In order to be able to work with fsQCA, a coding process called “calibration” [59] is first carried out, which consists of calculating the degree of belonging of each case both in its causal conditions and in its outcome. Thus, the responses are calibrated considering three thresholds for calibrating an original scale into a fuzzy set scale [56]: 5% percentile (low agreement or totally out of the category), 50% percentile (intermediate level of agreement or neither in nor out of the category) and 95% percentile (high agreement or totally in the category). Table 2 provides the original values for these three points for each of the four independent variables and job performance. In this process, the fuzzy condition for each condition has been obtained by naming the original variable code and adding “fs”, from the fsQCA function: f_cod = Calibrate (cod, full, mid, non-full). Cod is the input causal condition for calibration, f_cod is the fuzzy causal condition, and full, mid, and non-full are the three values that define the fuzzy set in the calibration process.

The model for analysis is:

perffs = f (leaderfs, commfs, satisfs, envirfs),

which explains what conditions are conducive to ensuring good job performance.

The first step is to examine the conditions necessary for the outcome. The consistency does not exceed Ragin’s [64] recommended cut-off value of 0.75 for any condition (see Table 3). Therefore, no single condition by itself ensures good performance. However, given the consistency values, the most important conditions even without being necessary are leadership and commitment.

The fsQCA method allows to analyse combinations of conditions (causal configurations) that lead to the output variable job performance. FsQCA calculates three possible solutions: complex, parsimonious and intermediate; the latter is the one that will be used, as suggested in the literature [59]. Table 4 presents the results of the intermediate solution. The symbol * represents the logical AND operator. The configurations connected by * are sufficient conditions to cause the result (i.e., performance). The symbol ~ represents the negation of the characteristic. The consistency of the analysis is 0.79 (above 0.74), so the model is informative [65] and there is a sufficient relationship between good job performance and a certain subset of conditions.

Table 5 shows the two combinations obtained (solutions 1: leaderfs * ~ satisfs * commfs; and 2: leaderfs * envirfs * commfs). Solution 2 reaches the maximum raw coverage and a great unique coverage (0.30). The other combination reaches 0.13 as unique coverage. Solution 2 is the most outstanding combination for solid performance.

## 4. Discussion

The discussion examines the two solutions resulting from the analysis. Ragin [59] recommends a consistency threshold of 0.80. All configurations meet this threshold.

The first configuration is leaderfs * ~ satisfs * commfs, which shows that a sufficient condition for a good level of job performance is a combination of leadership and commitment on the part of the most dissatisfied or least satisfied workers. For those who are dissatisfied, it makes no difference whether there is a good or bad work climate, what does not make a difference is that for good performance to occur there has to be leadership and commitment. Satisfaction by itself does not necessarily lead to better performance. This finding is in line with other authors such as Brayfield and Crocket [28] and Spencer and Steers [29]. On the contrary, dissatisfaction can be compensated by leadership and commitment to ensure performance.

The second configuration contains the variables leaderfs * envirfs * commfs, i.e., the presence of leadership, perception of a good work climate and employee commitment are sufficient conditions to ensure a good level of job performance.

These results allow us to contrast the four propositions that emerge from the literature review. All four propositions are confirmed, except in the case of the least satisfied workers, for whom the work climate is indifferent to improving their performance (proposition 4).

These findings have theoretical and practical implications. For research and theory, first, an innovative methodology is applied in the analysis of healthcare management: the fsQCA; second, an integrated model of job performance, leadership, organisational commitment, job satisfaction, and work environment is proposed. For practices and management of healthcare workers in times of crisis, we show that leadership and commitment are the key variables to ensure a good level of job performance in the case of less satisfied workers, and, in general, that there is a good working environment, in addition to leadership and commitment. It implies that if supervisors exercise good leadership they can create positive organisational commitment and further promote the job performance of healthcare professionals, in line with what other authors have argued [66,67]. In this regard, some of the actions that healthcare managers can take include, among others: (a) that management stimulates change and the implementation of a culture of trust, involvement and support oriented towards achieving best practices, as well as improving environmental conditions; (b) that integration of objectives is encouraged, with no barriers between individuals and/or groups/areas; (c) that continuous improvement is proactively pursued before having to react to conflicts or crises; (d) that the ideas of the members of the organisation are taken into account in the management of the organisation; (e) that environmental protection issues are proactively managed; (f) that employees feel a sense of pride in being part of the organisation; (g) that the values of the organisation are felt as their own by the employees; (h) or provide training programmes to cultivate the leadership talents of its employees. The aim will be to correct and enhance aspects that have been identified as “weak” and to maintain and/or improve other aspects identified as “strong”. Furthermore, in line with what is supported by other authors such as Rowe et al. [4], we recommend that ministries of health and international organisations should actively help translate research findings into action to improve health-worker performance.

The limitations of this research encourage the continuation of future lines of research. For example, the timing of the measurement of the study variables, during the COVID-19 pandemic, and the focus on the healthcare sector especially under extraordinary care pressure and workload, has influenced the results. The data is unique as it was collected in a specific situation during the pandemic [68]. We call for similar studies to be conducted in a time of normality and for their results to be compared. Finally, although fsQCA allows working with small samples and overcomes some limitations of the dominant research logic, i.e., cross-sectional surveys, single reports, self-reports, Likert scales, multiple regression analysis, structural equation techniques and models, we recommend extending the study with a larger representative sample to avoid sampling bias.

## 5. Conclusions

This research study examines the conditions (leadership, organisational commitment, job satisfaction, and work environment) that affect healthcare workers’ performance during COVID-19 times. The analysis employs fsQCA to identify the combinations of causes that lead to good job performance among healthcare professionals.

The results confirm proposition 1 (Leadership affects job performance). Moreover, leadership is present in both configurations, implying that in order to ensure good job performance, leadership is necessary [69]. Similarly, proposition 2 (Job performance improves with organisational commitment) is confirmed, i.e., for a good level of job performance, employee commitment (present in both configurations) is necessary in combination with other variables, including necessarily leadership. Employee commitment demonstrates an employee’s love for a specific work [70]. Additionally, healthcare workers have more than demonstrated an excellent commitment during the pandemic. Proposition 3 (Job satisfaction does not ensure better job performance) is also confirmed, i.e., satisfaction by itself does not necessarily lead to better performance. On the contrary, non-satisfaction can be compensated by leadership and commitment to ensure performance. The analysis does not confirm proposition 4 (Work climate influences job performance), since in one of the configurations (for the case of the least satisfied workers) the perception of a good work climate is indifferent for achieving good job performance. That is, for the most dissatisfied workers the work climate is not a relevant condition for their performance. However, the other configuration through leadership and commitment, the perception of a good working climate, is a necessary condition in the case of dissatisfied workers.

Leadership and commitment are the key variables to ensure a good level of job performance. The data confirm that the combination of leadership, commitment and a good work climate leads to good levels of job performance. Additionally, in the case of less satisfied workers, linking leadership and commitment is a sufficient condition.

Therefore, the contribution of this work is twofold. On the one hand, it applies an innovative methodology in the analysis of health management: fsQCA, which combines fuzzy logic with qualitative comparative analysis. Unlike conventional statistical techniques, fsQCA overcomes the limitations related to sample size and is less restrictive than case study methodology. This means that generalisation of conclusions and extrapolation of results to larger populations is possible [71]. On the other hand, the joint causal effects of key human resource variables are analysed to improve the job performance of health workers under conditions of care pressure.

## Figures and Tables

**Table 1:** Description of the respondent sample (*n* = 40).

		Frequency	Percentage
Age average = 49.28 (SD = 9.89; min. = 22; max. = 64)			
Gender			
	Male	13	32.5
	Female	27	67.5
Education level			
	Did not complete high school	1	2.5
	High school diploma/General Equivalency Diploma	12	30.0
	Bachelor’s degree	17	42.5
	University degree	10	25.0
Classification Statutory Staff of			
	Health	31	77.5
	Non-health (Management and Services)	9	22.5
Professional category			
	Administrative	1	2.5
	Hospital orderly	8	20.0
	Auxiliary nurse/Technician in Auxiliary Nursing Care	6	15.0
	Nurse	16	40.0
	Occupational therapist	8	20.0
	Medical Specialist in Physical Medicine and Rehabilitation	1	2.5
Responsibility/management			
	No position	36	90.0
	With position (unit supervisor, staff doctor, etc.)	4	10.0
Duration of employment contract			
	Permanent	32	80.0
	Long-term temporary	5	12.5
	Short-term temporary	3	7.5
Work hours			
	Full-time	40	100.0
	Part-time	0	0.0
Shift work			
	Fixed shift	18	45.0
	Rotating shift	22	55.0

SD = Standard Deviation.

**Table 2:** Summary data for job performance, leadership, organizational commitment, job satisfaction, and work environment study.

Statistics		Perf	Leader	Comm	Satis	Envir
*n*	Valid	40	40	40	40	40
	Missing	0	0	0	0	0
Mean		5.49	4.12	5.34	5.38	4.79
Std. error of mean		0.14	0.18	0.17	0.19	0.21
Median		5.25	4.50	5.58	5.67	5.07
Std. deviation		0.92	1.80	1.09	1.20	1.31
Calibration values at						
	95%	6.99	6.99	6.67	6.99	6.64
	50%	5.25	4.50	5.58	5.67	5.07
	5%	4.00	1.02	2.73	2.88	2.44

Perf = job performance; leader = leadership; comm = organizational commitment; satis = job satisfaction; envir = work environment.

**Table 3:** Analysis of necessary conditions (outcome variable: perffs).

	Consistency	Coverage
leaderfs	0.73	0.63
commfs	0.72	0.62
satisfs	0.59	0.55
envirfs	0.64	0.56

**Table 4:** Results of the intermediate solution (outcome: perffs).

Causal Configuration	Raw Coverage	Unique Coverage	Consistency
Leaderfs * ~ satisfs * commfs	0.41	0.13	0.82
Leaderfs * envirfs * commfs	0.64	0.30	0.86
solution coverage: 0.69			
solution consistency: 0.79			

**Table 5:** Configurations for achieving a good job performance.

Configuration	Solutions	
	1	2
Leadership	●	●
Organizational commitment	●	●
Job satisfaction	⊗	
Work environment		●
Raw coverage	0.41	0.64
Unique coverage	0.13	0.30
Consistency	0.82	0.86
Overall solution coverage:	0.69	
Overall solution consistency:	0.79	

● Core causal condition presence, ⊗ Core causal condition absence.

## Data Availability

The data presented in this study are available on request from the corresponding author. The data are not publicly available due to requirements of ethics approval.

## Appendix A

**Table A1:** Sources of the validated scales and Cronbach’s alpha reliability results in the current study.

Variable	Number of Items	Source/Validated Scale By	Reliability Cronbach’s Alpha
Job performance	4	Vandenabeele [7]	0.83
A sample item for job performance: In my opinion, I contribute to the success of the organization			
Leadership	6	Samson and Terziovski [44]	0.89
A sample item for leadership: Senior Managers actively encourage change and implement a culture of trust, involvement and commitment in moving towards ‘Best Practice’			
Organizational commitment	6	Benkhoff [46]	0.81
A sample item for organizational commitment: I am willing to put in a great deal of effort beyond that what is normally expected in order to help this organization to be successful			
Job satisfaction	6	Depré and Hondeghem [45]	0.87
A sample item for job satisfaction: In general, I am satisfied with my job			
Work environment	82	Patterson et al. [47]	0.88
A sample item for work environment: There is very little conflict between departments here			
